# Therapeutic potential of luteolin in central precocious puberty: insights from a danazol-induced rat model

**DOI:** 10.3389/fendo.2025.1666932

**Published:** 2025-09-12

**Authors:** Zhijian Zha, Enze Lei, Xiaofan Wu, Siyu Bai, Tiantian Huang, Tao Lu, Zifeng Wang, Yan Cai, Hui Li, Yao Chen, Jianzhong Liu

**Affiliations:** ^1^ College of Traditional Chinese Medicine, Hubei University of Chinese Medicine, Wuhan, China; ^2^ Department of Integrated Traditional Chinese and Western Medicine, Wuhan Children’s Hospital, Tongji Medical College, Huazhong University of Science and Technology, Wuhan, China; ^3^ Department of Traditional Chinese Medicine, Hubei Provincial Maternal and Child Health Hospital, Wuhan, China; ^4^ Department of Pediatrics, Hubei Provincial Hospital of Traditional Chinese Medicine, Affiliated Hospital of Hubei University of Chinese Medicine, Wuhan, China; ^5^ Hubei Shizhen Laboratory, Wuhan, China; ^6^ Hubei Key Laboratory of Liver and Kidney Research and Application of Traditional Chinese Medicine, Wuhan, China

**Keywords:** children, luteolin, danazol, precocious puberty, transcriptomics, reverse transcription- quantitative polymerase chain reaction

## Abstract

**Background:**

Recently, central precocious puberty (CPP) is becoming a major public health concern worldwide due to its enhanced prevalence. Traditional Chinese medicine (TCM) compounds offer unique therapeutic advantages for treating this condition, and luteolin, a bioactive monomer compound commonly found in these herbs, has drawn increasing attention. However, the therapeutic effects of luteolin on CPP development remain unclear.

**Methods:**

A danazol-induced CPP model was established in Sprague-Dawley rats to explore the potential therapeutic effects of luteolin. Sexual development indicators, organ coefficients, gonadal histopathology, and sex hormone levels were evaluated to assess treatment outcomes. Additionally, a comprehensive approach involving network pharmacology, molecular docking, and transcriptomic analyses was used to identify luteolin-related signaling pathways and target proteins involved in CPP treatment. Finally, we carried out enzyme-linked immunosorbent assay (ELISA) and reverse transcription- quantitative polymerase chain reaction (RT-qPCR) for finding validation and exploring the underlying mechanisms.

**Results:**

In the danazol-induced CPP model, luteolin treatment significantly decreased the abundances of Estradiol (E2), luteinizing hormone serum, and follicle-stimulating hormone in sera; reduced organ coefficients and ovarian and uterine wet weights; and delayed vaginal opening. Network pharmacology and transcriptomic analyses revealed that luteolin exerted its therapeutic effects mainly by modulating immune and inflammatory pathways, including the tumor necrosis factor-α, Toll-like receptor, and IL-17 signaling pathways. Molecular docking demonstrated stable binding of luteolin to key targets such as Cxcl10, Cxcl11, Stat1, Tlr3, and Irf7. ELISA results confirmed that luteolin inhibited pro-inflammatory cytokines while promoting anti-inflammatory factors in the CPP model. Furthermore, RT-qPCR analysis revealed that luteolin enhanced Irf7 and Stat1 expression within the Toll-like receptor pathway, mainly by upregulating Tlr3, thereby enhancing the abundances of downstream effector molecules Cxcl10 and Cxcl11.

**Conclusion:**

This study is the first to determine that luteolin ameliorates CPP via the Toll-like receptor signaling pathway. These findings enhance our understanding of luteolin’s pharmacological actions and support its potential role in CPP treatment.

## 1 Introduction

The global incidence of precocious puberty (PP), a common perdiatric endocrine disorder, has increased steadily over the past 25 years (1, 2). By classical definition, PP refers to premature puberty initiation at ages of <8 and 9 years respectively for females and males. PP not only compromises final adult height owing to accelerated bone maturation but also increases the risk of metabolic, reproductive, and psychological disorders in affected children (3, 4). The onset of PP is closely associated with environmental, dietary, and nutritional factors (5). Notably, these factors can directly activate hypothalamic orexin signaling, induce an inflammatory state, and subsequently trigger premature gonadotropin-releasing hormone (GnRH) neuron activation, leading to central PP (CPP) development (6–8). Based on etiology, precocious puberty can be classified into three types: central precocious puberty, peripheral precocious puberty, and incomplete precocious puberty, approximately 80% of PP cases are classified as CPP (9). Although GnRH analogs (GnRHa) are recommended as the standard treatment for CPP (10), they have strict indications and contraindications and are associated with high costs (11). Therefore, identifying novel therapeutic alternatives that are both effective and safe for CPP is of paramount importance.

Traditional Chinese medicine (TCM) formulations have shown promising potential in CPP treatment, offering a multi-targeted therapeutic approach (12). For example, compounds such as “Zhibai Dihuang Pill” and “Fuyou Formula,” both rich in luteolin, have demonstrated clear efficacy and a favorable safety profile in managing CPP (13, 14). The flavonoid luteolin is abundant in various fruits, vegetables, and several Chinese herbal medicines. High concentrations of luteolin are present in dietary sources such as celery, chili peppers, lettuce, spinach, and thyme, as well as in medicinal herbs including perilla (Perilla frutescens) leaves, Origanum vulgare, and Juniperus communis (15).It is known to regulate multiple cellular signaling pathways potentially involved in endocrine function. This suggests its potential role in rebalancing the dysregulated hypothalamic-pituitary-gonadal axis (HPGA) observed in CPP (16). Moreover, luteolin exhibits anti-inflammatory, antioxidant, and neuroprotective properties (17). Despite its therapeutic promise, the precise interventional effects and underlying mechanisms of luteolin in the context of CPP remain inadequately understood.

For evaluating luteolin’s efficacy during CPP treatment and investigating the underlying biological pathways, we designed this research. A danazol-induced CPP model was first established in Sprague-Dawley (SD) rats to assess the efficacy of luteolin treatment, administration of danazol during the neonatal period disrupts the feedback mechanisms of sex hormones and neuropeptide networks during the critical developmental window of the hypothalamus, prematurely activating the HPGA. This leads to an early increase in LH/FSH and estradiol levels, resulting in a precocious puberty phenotype in female rats (18, 19). Network pharmacology and transcriptomics, both widely applied in the study of TCM, were employed to elucidate the molecular mechanisms involved. As a method of unveiling the complex interactions between TCM effective compounds and their target proteins, the utility of molecular docking and network pharmacology analyses has been validated (20). Transcriptomics offers in-depth insights into gene expression patterns and regulatory networks, enabling the identification of key pathways modulated by TCM interventions (21). Utilizing these techniques in a comprehensive manner, we herein identified the key gene expression changes and major signaling pathways influenced by luteolin during CPP treatment (22). Finally, the transcription and secretion levels of specific signaling molecules were appraised for result verification. Collectively, our findings reveal novel comprehension supporting the utilization of luteolin for CPP management.

## 2 Materials and methods

### 2.1 Drugs and reagents

Livzon Pharmaceutical Group (Zhuhai, China), A&D Technology Corporation (Beijing, China), and National Institutes for Food and Drug Control (Beijing, China) were the respective providers for leuprorelin acetate microspheres, danazol, and TCM reference standard luteolin (94.40% purity, 111520-202006), Chemical formula: C15H10O6; molecular weight: 286.24 g86.24:a chemical structure shown in **Supplementary Figure 1**. Pentobarbital sodium (Sigma-Aldrich, USA) was used as the anesthetic agent. All remaining chemicals employed herein met ultra-pure specifications.

### 2.2 Animal grouping and drug administration

Rat dams and the corresponding female offspring were acquired from the Animal Center of Three Gorges University on postnatal day (PND) 3. Housing conditions included evenly divided illuminated–non-illuminated scheme and unlimited food and water supply. At PND 21, the pups were weaned and separated from their mothers. All experimental procedures conformed to the Helsinki Declaration and received approval from the Animal Experimental Center of Hubei University of Traditional Chinese Medicine (approval number: HUCMS00311960).

**Figure 1:**
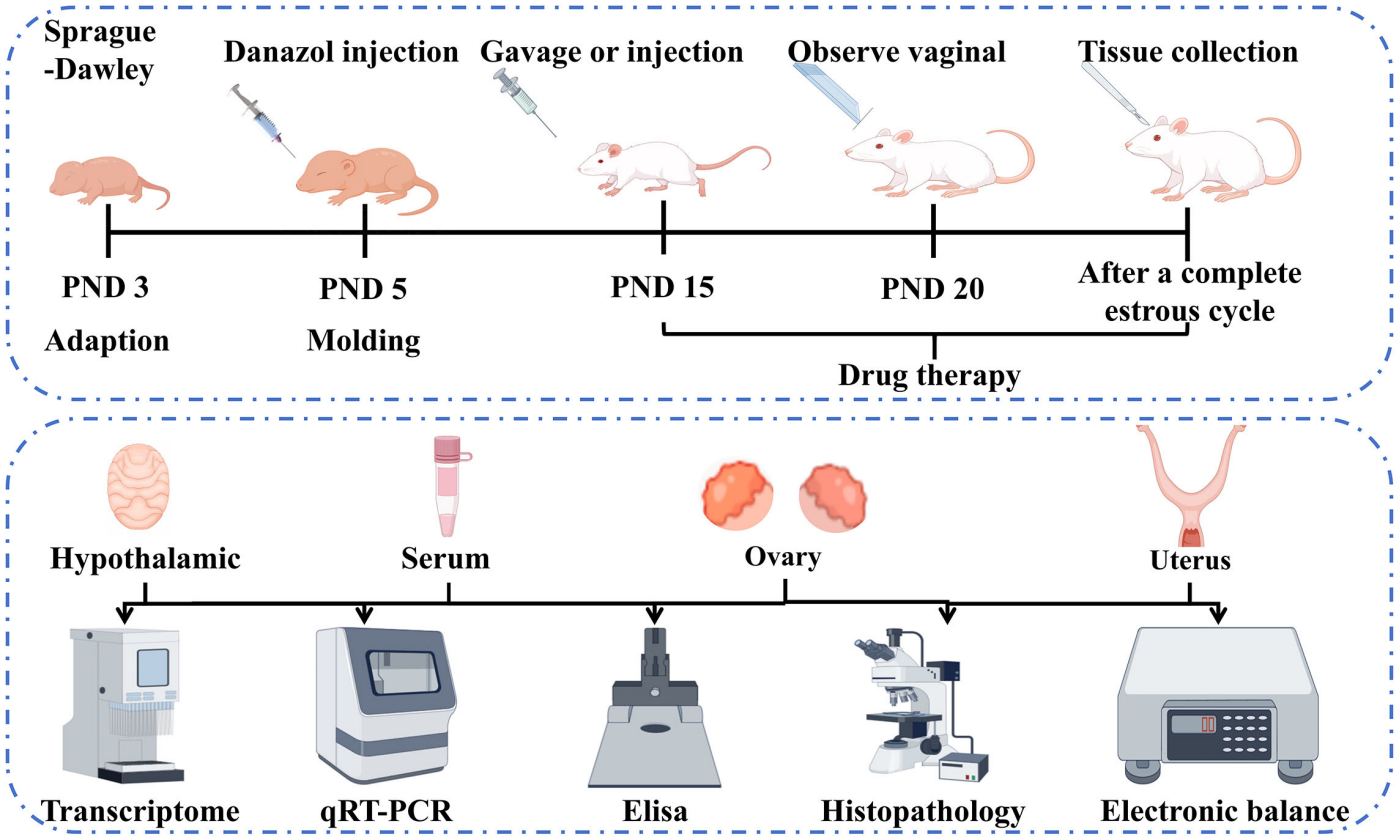
Flowchart outlining the *in vivo* experimental design and assay for this study.

The animals were randomly assigned to five groups, namely control, model, triptorelin (positive control), luteolin high-dose (HD), and luteolin low-dose (LD) groups, with 6 rats per group. On PND 5, 300 µg/25 µL of danazol (in a 1:1 mixture of ethylene glycol and ethanol, v/v) was administered to the rats in the model, triptorelin, and luteolin groups via subcutaneous injection. The control group received 25 µL of the glycol/ethanol vehicle alone (19, 20). Starting on PND 15, the triptorelin group was administered 100 μg/kg of triptorelin via subcutaneous injection. The luteolin LD and HD groups were intragastrically administered 60 and 100 mg/kg of luteolin, respectively, while equal volumes of physiological saline were provided to the rats not subjected to luteolin or triptorelin treatments via intragastrical administration. From PND 20 onward, a vaginal opening was monitored and recorded daily as an indicator of pubertal onset. Following one full estral cycle, rats exhibiting open vagina were subjected to diestrous euthanization, while the rest were sacrificed at a corresponding phase of the cycle. The specific procedure is as follows: Rats were anesthetized by intraperitoneal injection of pentobarbital sodium (50 mg/kg). After ensuring that the rats were in a state of deep anesthesia, the blood of abdominal aorta was collected. After the blood collection was completed, euthanasia was performed in strict accordance with the AVMA Guidelines for the Euthanasia of Animals (2020 Edition). Use the thumb and index finger to press down on the head and neck, while the other hand grasps the tail or hind limbs. Quickly and forcefully pull the hindquarters backward and upward to dislocate the cervical vertebrae. Check the animal’s heartbeat and the pupils to confirm death. Hypothalamic tissues harvested meticulously from sacrificed rats were snap-frozen with LN2 and kept inside the -80°C freezer. The uterus and ovaries were dissected for weight measurement to calculate organ coefficients. Hematoxylin and eosin (H&E) staining was carried out utilizing half of the tissues, which were subjected to fixation within paraformaldehyde (4%), with the rest utilized for other analyses being kept inside the -80°C freezer. Besides, Abdominal aortic blood was centrifuged (3,500 rpm, 20 min, 4°C) for separation of serum samples. The prepared sera were kept inside a -80°C freezer (**Figure 1**).

### 2.3 Histopathological analysis

Fixed ovarian and uterine tissues were dehydrated and paraffin embedded. The embedded tissues were sectioned (5 μm) and then sequentially subjected to xylene deparaffinization and rehydration with graded decreased concentrations of ethanol in water. H&E staining was performed on all sections. After staining, images were captured using a Nikon Eclipse C1 digital camera connected to a Nikon DS-FI2 digital microscope (Nikon, Japan). Uterine tissue sections were imaged at 100× magnification. Endometrial thickness was measured (in mm) at five different locations on each slide employing Image-Pro Plus 6.0 for calculating the average value. For ovarian tissue, the number of corpora lutea was counted on each slide.

### 2.4 Enzyme Linked Immunosorbent Assay detection

The serum abundances of Estradiol (E2), luteinizing hormone (LH), and follicle-stimulating hormone (FSH) were measured using commercially available ELISA kits (Cusabio Biotech Co., Ltd., Wuhan, China). For each assay, 50 µL of rat serum was added to each sample well.

The abundances of tumor necrosis factor-α (TNF-α),Interleukin 4 (IL-4), Interleukin 17 (IL-17), Interleukin 10 (IL-10) and Interleukin 6 (IL-6) were assessed using ELISA kits from Elabscience Biotechnology Inc. (Wuhan, China). Protein extracts from hypothalamic and ovarian tissues were analyzed. Cytokine levels in tissue homogenates were normalized to total protein levels determined through bicinchoninic acid assay quantification. For the analysis of ovarian tissue, 40 µL of rat serum was added to each sample well.

All ELISA procedures were performed according to the manufacturer’s protocols. Hormone and cytokine measurements were performed following the recommended guidelines to ensure accuracy and reproducibility.

### 2.5 Network pharmacology analysis

An extensive search of the databases of OMIM (https://omim.org/), DisGeNET (https://www.disgenet.org/), and GeneCards (https://www.genecards.org/) led to the identification of 2,603 CPP-associated Homo sapiens targets, among which 2,383 unique targets remained after deduplication. Potential luteolin targets were retrieved from the SwissTargetPrediction (http://swisstargetprediction.ch/), PharmMapper (http://www.lilab-ecust.cn/pharmmapper/), Sea (https://sea.bkslab.org/), and TCMSP (http://tcmspnw.com/) databases, resulting in 147 non-redundant targets. Two target networks, one for CPP and one for luteolin, were constructed using Cytoscape v3.10.0 and the stringApp plugin. The overlap between the two networks was assessed, and key nodes were identified based on degree values exceeding the average, indicating their potential importance in the interaction network. The R (v4.0.1) package DESeq2 (v1.30.0) was employed for screening genes displaying differential expression (DEGs), with the cutoff fold change and adjusted p-value values respectively being 1.5 and 0.05. Afterwards, clusterProfiler (v3.18.1) was utilized for Kyoto Encyclopedia of Genes and Genomes (KEGG) and Gene Ontology (GO) functional characterization of the DEGs.

### 2.6 Molecular docking studies

For simulating the type and strength of luteolin-protein interactions in silico, we employed the AutoDock Vina 1.2.2 (http://autodock.scripps.edu/) software (23). PubChem Compound (https://pubchem.ncbi.nlm.nih.gov/) and RCSB Protein Data Bank (http://www.rcsb.org/pdb/home/home.do) were respectively queried for acquiring the stereochemical structures of luteolin and its target molecules Cxcl10 (PDB ID: 1O80), Cxcl11 (1RJT), Irf7 (2O61), Stat1 (1YVL), and Tlr3 (1ZIW) (24). Prior to the simulations, PDBQT files were created for all the aforementioned molecules. To improve docking accuracy, water molecules were replaced with polar hydrogen atoms. The grid box (30 × 30 × 30 Å, 0.05-nm spacing) for each target protein was centered over the active domain and to ensure sufficient space for free molecular movement.

### 2.7 Transcriptome profiling

TRIzol (Invitrogen) was employed for isolation of total RNA from hypothalamic tissues, which was subjected to DNase I (Takara) treatment (25). For quantifying and assessing the integrity of RNA, 1% agarose gel electrophoresis, spectrophotometry with NanoDrop 2000, and automated electrophoresis with the Agilent 2100 Bioanalyzer system were carried out. Differential gene expression analysis for samples with biological replicates was conducted using the DESeq R package (v1.10.1) to identify DEGs (adjusted *p*-value < 0.05). For samples without biological replicates, read counts were normalized using a scaling factor provided by the edgeR R package, with significance defined as Q-value of < 0.005 and |log2(fold change)| ≥ 1. Additionally, DESeq2 (v1.30.0) was used to conduct differential expression analysis, with significance thresholds set at an adjusted *P*-value < 0.05 and fold change ≥ 1.5. Finally, ClusterProfiler (v.3.18.1) was adopted for GO and KEGG functional characterizations of the DEGs (26, 27).

### 2.8 Reverse transcription- quantitative polymerase chain reaction assays

The TaKaRa RNAiso kit (Cat No. 9109) was adopted for isolation of hypothalamic tissue total RNA, with its quality and quantity being appraised with the spectrophotometric and gel-electrophoretic methods mentioned above. The PrimeScript™ RT Reagent Kit (TAKARA, RR037A) was adopted for reversely transcribing one microgram RNA sample into cDNA through a 15-min incubation at 37°C, with the reaction being terminated by a 5-s heating step at 85°C. Afterwards, 2 µL of the cDNA and 0.5 µM primer pairs (synthesized by Beijing Qingke) were assembled with the YEASEN Hieff™ qPCR SYBR Green Master Mix (No Rox) (Cat No. 11201ES08) into a reaction system as specified by the manufacturer. Thermocycling initiated with a 3-min denaturation step at 95°C. Forty subsequent cycles involved 10 s at 95°C, 0.5 min at 60°C, and 0.5 min at 72°C. Specificity of primers was confirmed via a melting curve analysis, for which the temperature was increased stepwise at a rate of 0.5°C/5 s from 65°C to 95°C. Primer sequences for target genes and reference genes (GAPDH or β-actin) are provided in the **Supplementary Material**. The abundances of target transcripts relative to those of internal reference genes were determined through the 2^–ΔΔ^Ct algorithm (28). **Table 1** details the sequences of RT-qPCR primers.

**Table 1:** Primer table.

Gene	5’→3’	5’→3’	PCR Products (bp)
GAPDH	TCTCTGCTCCTCCCTGTTC	ACACCGACCTTCACCATCT	87
Irf7	GCAAGAGGAAATGCTGGGTTG	TAGCTTCCATCTGCCATGCT	196
Cxcl10	TGAAAGCGGTGAGCCAAAGA	CTAGCCGCACACTGGGTAAA	129
Stat1	GAGAGGTCTCAACGCTGACC	CACCCATCATTCCAGAGGCA	198
Tlr3	TCACTTCGAGGGTTGGAGGA	TGCCGTGTATTCGAACTGCT	106
Cxcl11	CCTGGCTATGATCATCTGGG	TTGTCACAGCCGTTACTCGG	150

### 2.9 Statistical analysis

Statistical analysis was conducted employing SPSS (v29.0) and GraphPad Prism (v8.02) software. Results are expressed in the form of mean ± standard deviation. Independent groups were comparatively analyzed in a pairwise manner employing unpaired t-tests or one-way analysis of variance with least significant difference *post-hoc* testing for two and more than two groups, respectively. Statistical significance was defined as *P* < 0.05.

## 3 Results

### 3.1 Luteolin inhibits danazol-induced CPP formation in SD rats

**Figure 2:**
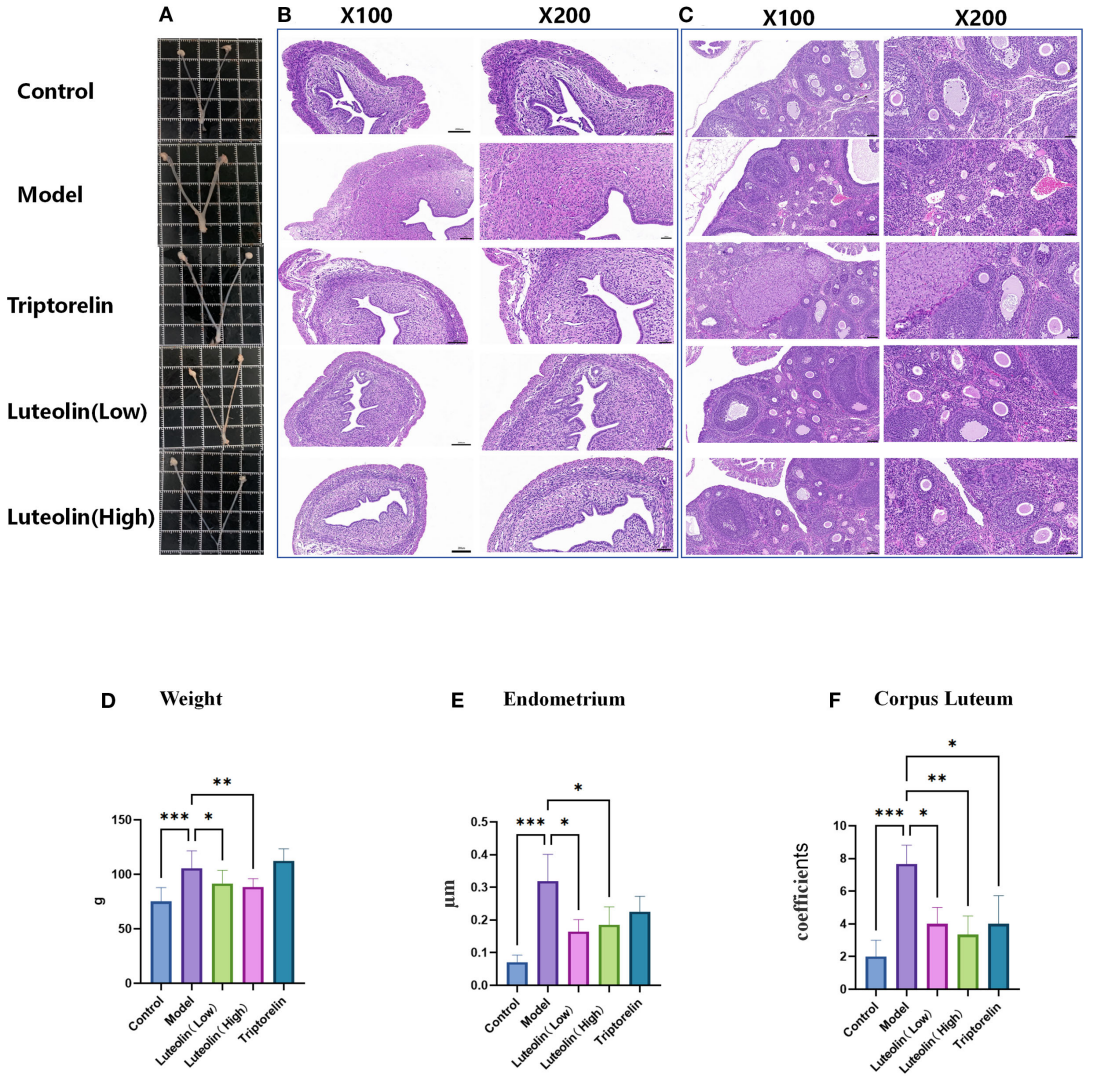
Effects of luteolin on uterine wall thickness and fallopian tubes. **(A)** Macroscopic view of bilateral ovaries and uterus. **(B)** Microscopic observation of uterine wall by HE staining (original magnification,100× and 200×). **(C)** Microscopic observation of Ovarian follicle were observed by HE staining (original magnification, 100× and 200×). **(D)** Luteolin can reduce weight. **(E)** Luteolin reduces the thickness of uterine wall. **(F)** Luteolin reduces the number of corpus luteum in ovary. *P<0.05; **P<0.01; ***P<0.001.

Organ sampling photographs showed that, following danazol administration, the fallopian tubes, uterus, and ovaries were significantly enlarged in the model group rats relative to the control counterparts. Conversely, the reproductive organs in the triptorelin and luteolin LD and HD groups were noticeably smaller compared to those in the model group (**Figure 2A**). H&E staining revealed that the model group exhibited a thickened endometrium, a reduced number of primary and secondary oocytes in the ovaries, and an increased number of mature oocytes and corpora lutea compared to the control group. In comparison, the triptorelin and luteolin LD and HD groups showed a reversal of these changes, with increased numbers of primary and secondary oocytes and reduced numbers of mature oocytes and corpora lutea (**Figures 2B,C**). We also analyzed the rats’ body weight and found that it increased evidently in the model group relative to all the other groups, except the triptorelin group, suggesting a regulatory effect of luteolin on body weight (**Figure 2D**). Statistical analysis of uterine wall thickness revealed that the model group had a significantly thicker uterine wall compared with all other groups, again with the exception of the triptorelin group (**Figure 2E**). Additionally, the model rats showcased increased number of corpora lutea relative to the controls, a change that was reversed by luteolin and triptorelin treatments (**Figure 2F**).

**Figure 3:**
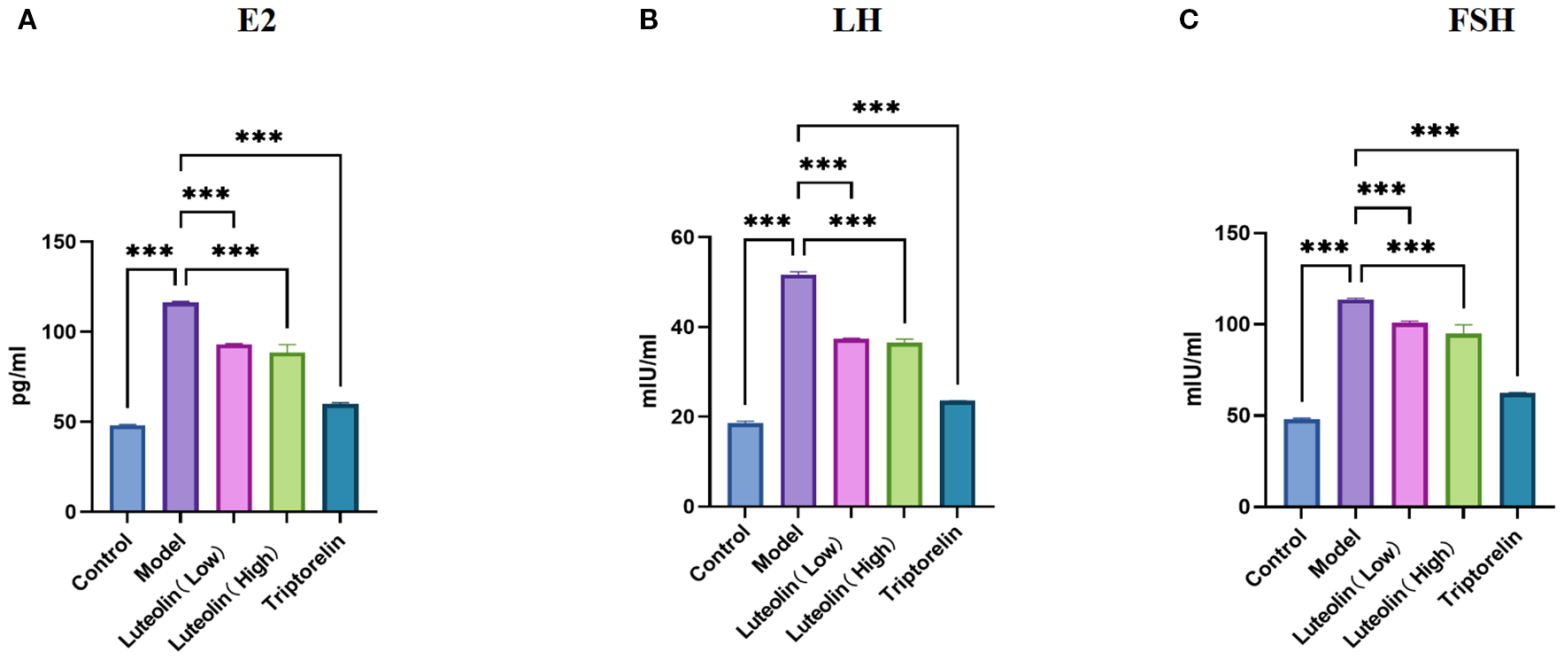
Effects of the luteolin on serum levels of the sex hormones. **(A–C)** Luteolin reduced the serum LH **(A)**, FSH **(B)**, and E2 **(C)** levels. ***P<0.001.

ELISA was used to measure serum estradiol, LH, and FSH levels. All three hormones exhibited a similar trend: the model group had significantly elevated E2, LH, and FSH levels relative to the controls. Conversely, hormone levels in both the luteolin-treated and triptorelin-treated groups were markedly reduced relative to the CPP rats. The findings reveal that danazol modeling accelerates sexual development by increasing sex hormone levels, while both luteolin and triptorelin interventions effectively suppress this effect (**Figure 3**). Furthermore, luteolin not only significantly inhibited sexual development in the modeled rats—exerting a therapeutic effect comparable to triptorelin—but also demonstrated a greater impact in reducing body weight.

### 3.2 Predictive network pharmacology

**Figure 4:**
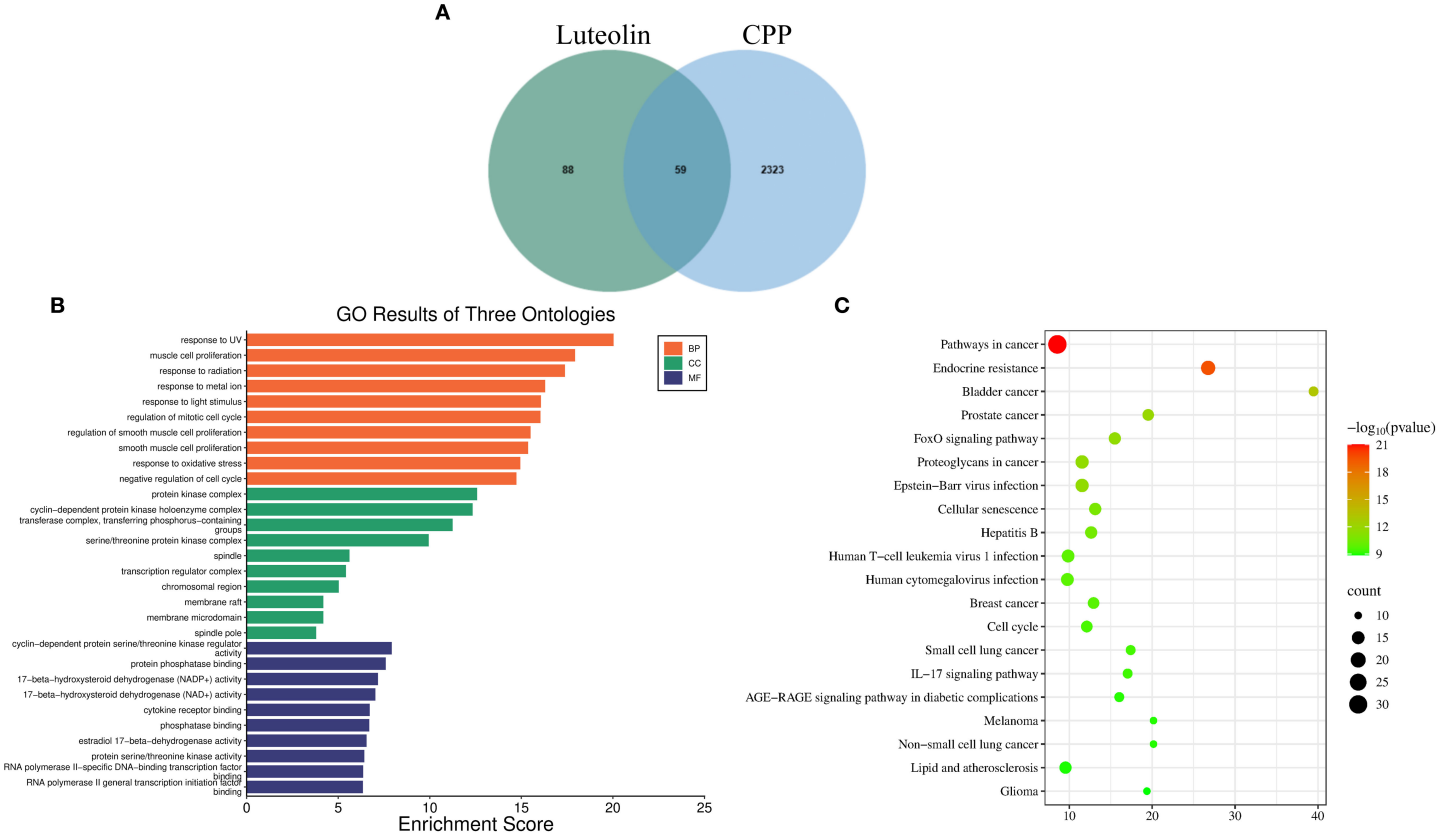
Network pharmacology results. **(A)** Venn diagram of intersecting targets of luteolin and CPP. **(B, C)** GO **(B)** and KEGG **(C)**, top 20 pathways, analyses of intersecting targets in network pharmacology.

Using network pharmacology based on TCM, we predicted the potential targets of luteolin and CPP. A total of 147 luteolin-related targets, 2,382 CPP-related targets, and 59 overlapping (intersection) targets were identified (**Figure 4A**). GO analysis indicated that luteolin may exert therapeutic effects on CPP through modulating the expression of genes exhibiting biological process, molecular function, and cellular component-associated functionalities (**Figure 4B**). KEGG pathway enrichment analysis revealed that, in addition to endocrine-related pathways such as endocrine resistance, luteolin was significantly associated with immune-related pathways. These included IL-17, T cell receptor, and Toll-like receptor signaling cascades, as well as Human cytomegalovirus infection, Hepatitis B, and Human T-cell leukemia virus 1 infection (**Figure 4C**).

### 3.3 Transcriptomic insights

**Figure 5:**
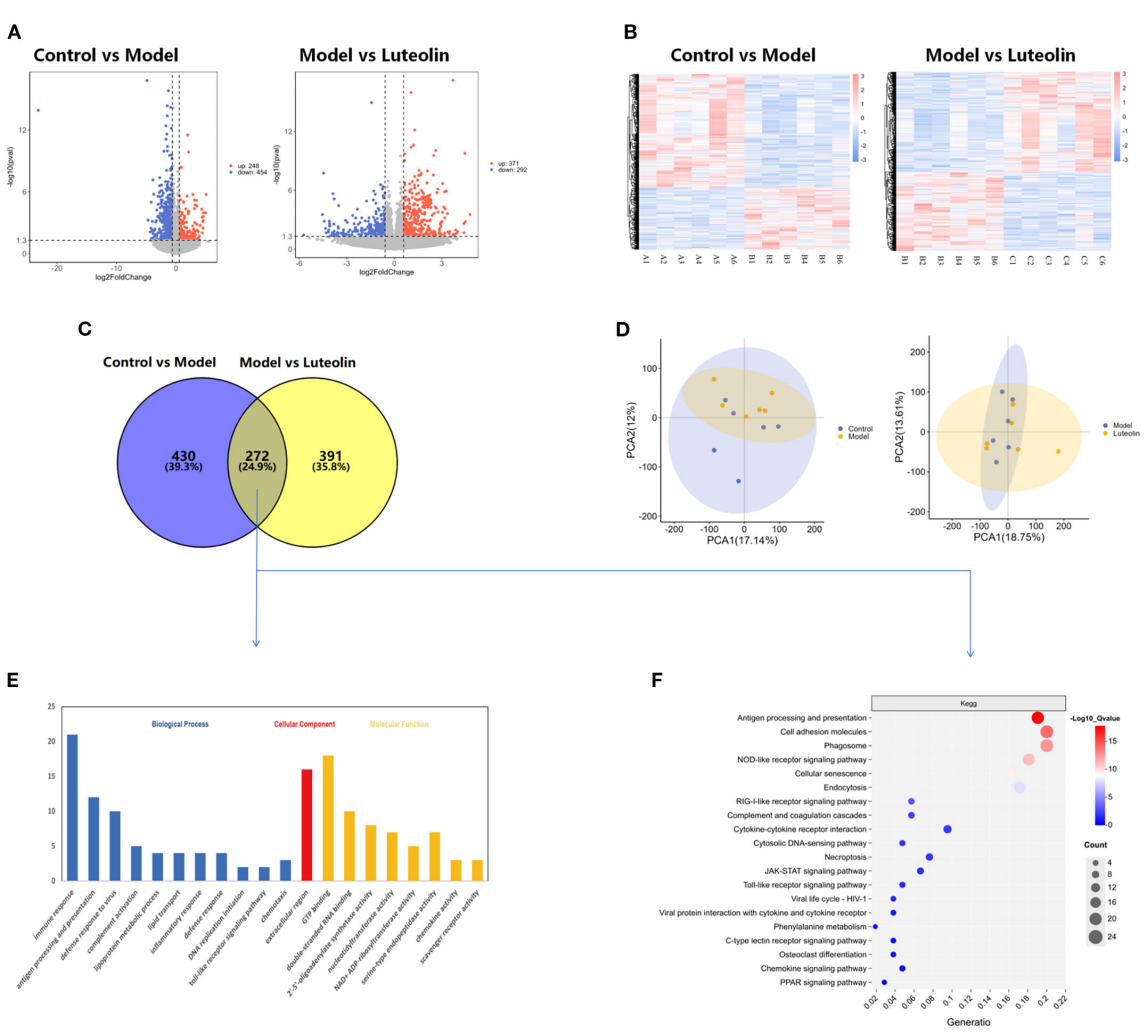
Transcriptomics results. **(A)** Volcano plot of differentially expressed genes between Control vs Model and Model vs Luteolin. **(B)** Clustering plot of differentially expressed genes between Control vs Model and Model vs Luteolin. **(C)** Venn diagram of differentially expressed genes between Control vs Model and Model vs Luteolin. **(D)** PCA plot of Control vs Model and Model vs Luteolin. **(E)** GO analysis plot of differentially expressed genes between Control vs Model and Model vs Luteolin. **(F)** KEGG analysis plot of differentially expressed genes between Control vs Model and Model vs Luteolin.

Transcriptomic analysis of hypothalamic tissue was performed to determine the molecular pathways influenced by danazol and luteolin treatments. The analysis led to the identification of 702 and 663 DEGs between the control group vs. model group and model group vs. luteolin-treated group comparisons, respectively. For the two cohorts of DEGs, 248 and 371 exhibited enhanced expression and 454 and 292 displayed reduced expression, respectively. By intersecting the DEGs from both comparisons, a total of 272 common DEGs were identified (**Figures 5A–C**). Subsequently, hierarchical clustering analysis was performed to group these DEGs based on their expression profiles across different samples. This analysis revealed distinct gene expression patterns, allowing for the identification of genes with similar regulatory behavior, involved in related signaling pathways or biological functions. **Figure 5B** illustrates that gene expression patterns are consistent within each group, while notable differences are observed between groups. To further characterize gene expression variations, we conducted principal component analysis using the expression data from individual samples. The principal components 1 (PC1) and 2 (PC2) were calculated to capture the major variance between samples, and the position of each sample was plotted as a distinct colored point on the coordinate axes. The spatial distances between points reflect the clustering relationships, indicating the degree of similarity or dissimilarity between samples (**Figure 5D**). Functional characterization of the 272 common DEGs was then carried out through KEGG and GO analyses. GO annotation reflected that the DEGs primarily exhibited enrichment with immunologic processes of immune response, inflammatory response, and antigen processing and presentation (**Figure 5E**). As for pathways, the DEGs predominantly displayed enrichment with NOD-like receptor, RIG-I-like receptor, Toll-like receptor, and JAK-STAT signaling cascades, as well as antigen processing and presentation, among others (**Figure 5F**), which are largely involved in immune-related functions. These findings support the hypothesis that luteolin may alleviate CPP by modulating immune and inflammatory signaling.

### 3.4 Integrative transcriptomic and network pharmacology analysis

**Figure 6:**
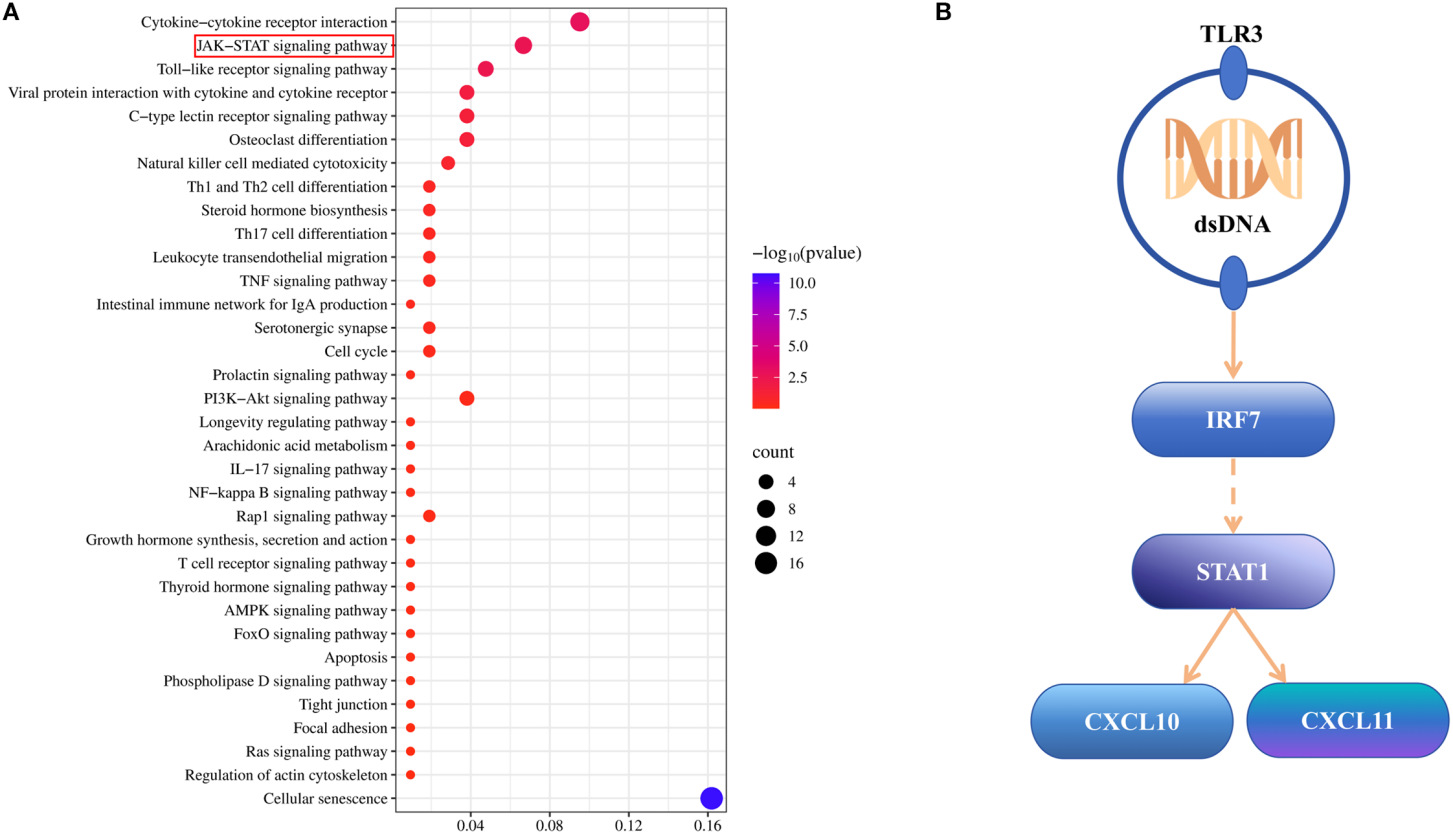
Integrated analysis results. **(A)** KEGG analysis diagram of integrated analysis, showing all identical KEGG pathways. **(B)** Gene upstream and downstream relationship diagram focusing on the toll-like receptor signaling pathway.

For further exploring the mechanistic insights into CPP’s therapeutic effects on CPP, we integrated KEGG pathway results from both network pharmacology and transcriptomic analyses. By identifying overlapping pathways between the two methods, we constructed a new KEGG enrichment profile based on their intersection, identifying 34 shared pathways (**Figure 6A**). Of these shared pathways, 14 were directly related to immune and inflammatory responses, including the C-type lectin receptor, TNF, prolactin, IL-17, NF-κB, T cell receptor, and Toll-like receptor signaling cascades, as well as the Toll pathway in humans, cytokine–cytokine receptor interaction, viral protein interaction with cytokine and cytokine receptor, Th1 and Th2 cell differentiation, Th17 cell differentiation, leukocyte transendothelial migration, and intestinal immune network for IgA production. According to this analysis, we focused on the Toll-like receptor signaling pathway as a representative inflammatory pathway. Transcriptomic analysis identified five DEGs involved in this pathway: Tlr3, Irf7, Stat1, Cxcl10, and Cxcl11. The upstream and downstream regulatory relationships among these genes are illustrated in **Figure 6B**.

### 3.5 Molecular docking

**Figure 7:**
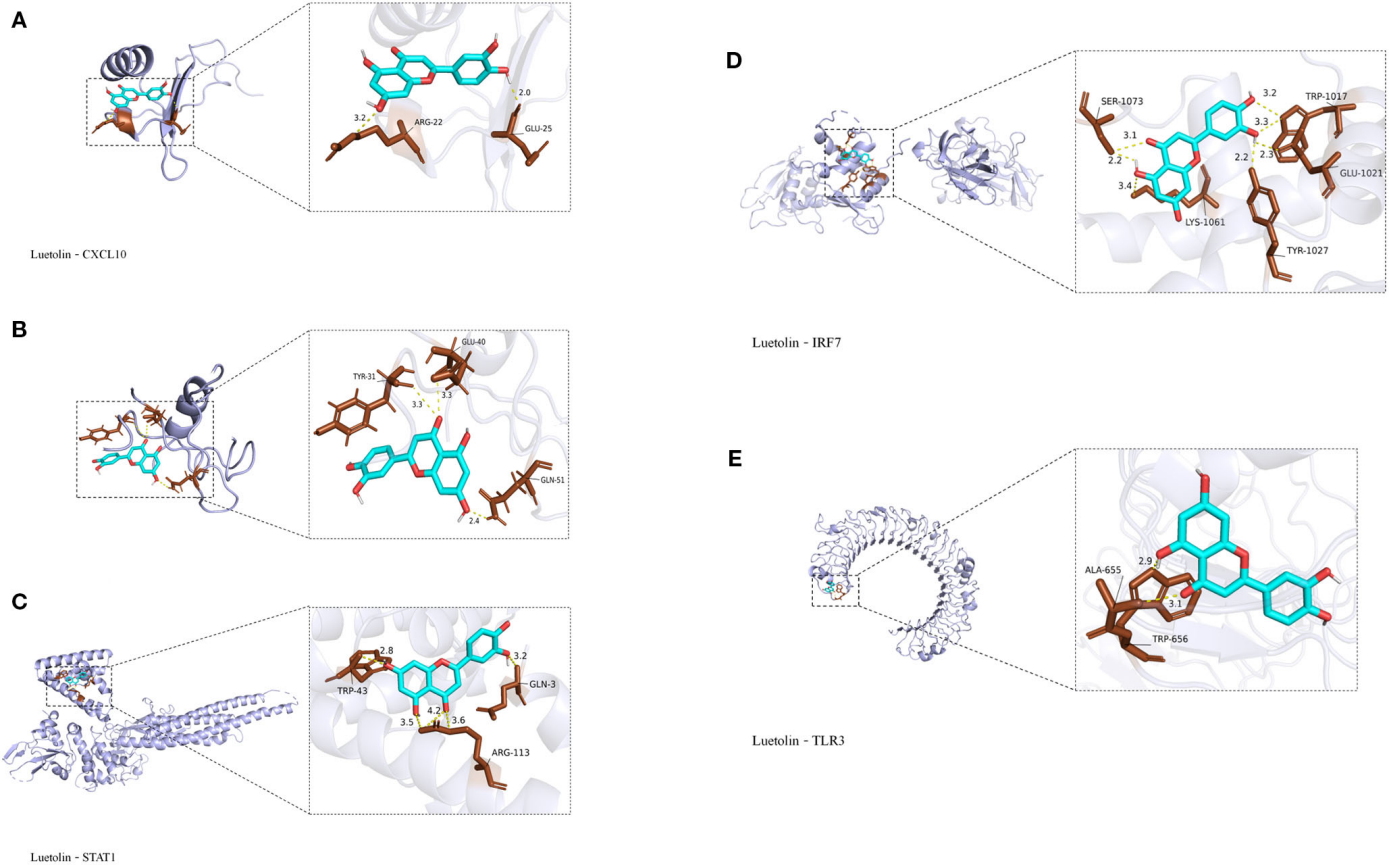
Molecular docking results. **(A)** Luteolin and Cxcl10 molecular docking. **(B)** Luteolin and Cxcl11 molecular docking. **(C)** Luteolin and Stat1 molecular docking. **(D)** Luteolin and Irf7 molecular docking; **(E)** Luteolin and Tlr3 molecular docking.

Molecular docking analysis using AutoDock Vina v1.2.2 was performed to examine the binding affinities between luteolin and its target proteins. The docking results provided the binding poses and interaction profiles of luteolin with five protein targets, and the corresponding binding energies were calculated. The analysis revealed that luteolin interacted with its targets through strong electrostatic interactions and visible hydrogen bonds (**Figure 7**). The binding energies were as follows: -6.1 kcal/mol for Cxcl10, -6.3 kcal/mol for Cxcl11, -7.1 kcal/mol for Stat1,-6.9 kcal/mol for Tlr3, and -7.1 kcal/mol for Irf7, suggesting stable and favorable binding interactions (**Table 2**).

**Table 2:** Molecular docking binding energy table.

Name	Affinity (kcal/mol)
Irf7	-6.3
Stat1	-7.1
Tlr3	-6.9
Cxcl11	-6.3

### 3.6 Luteolin inhibits inflammatory reaction

**Figure 8:**
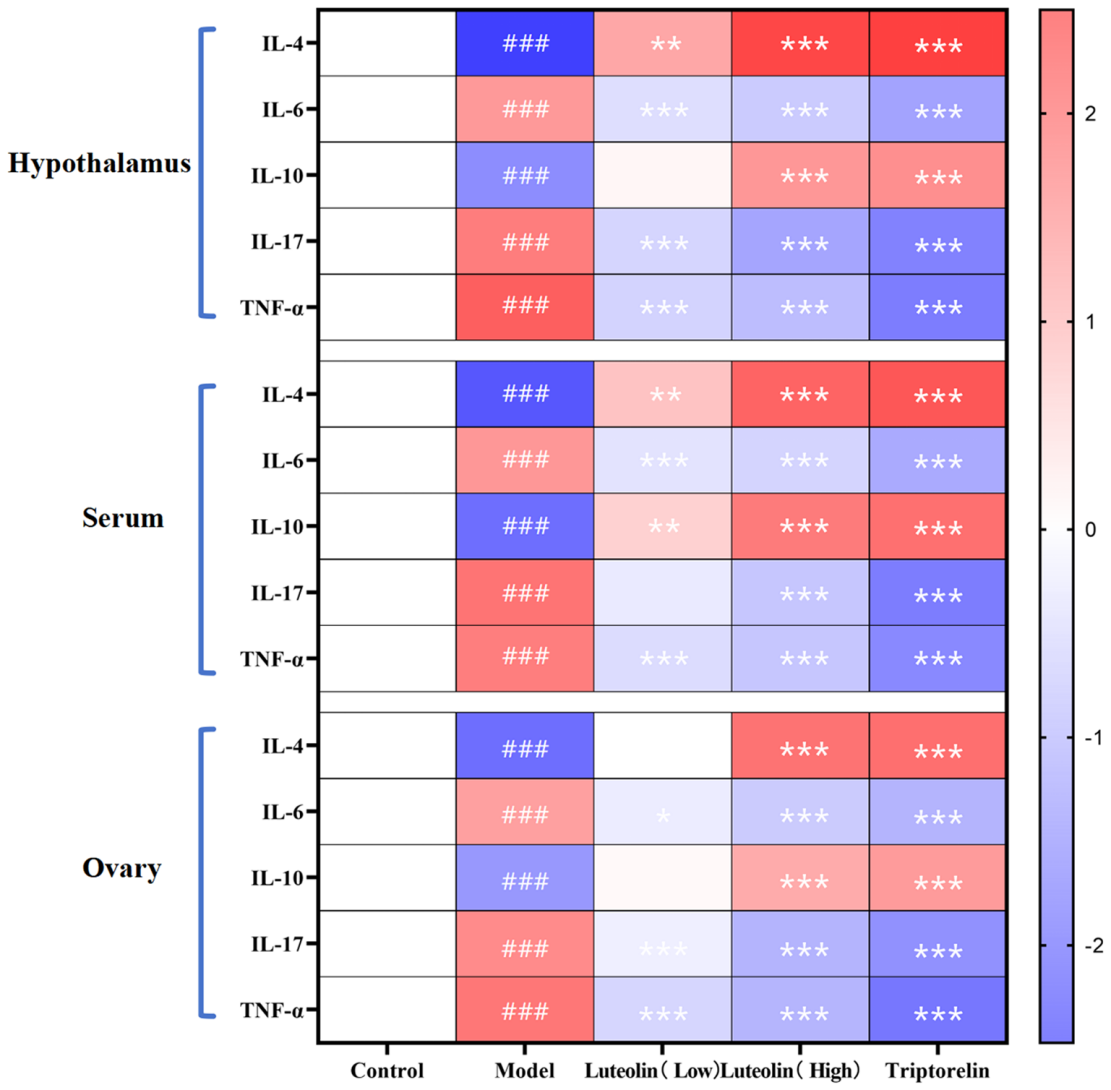
Immune factor detection results heat map. ###*P*<0.001 compared to the control group; and *P<0.05; **P<0.01; ***P<0.001 compared to the model group. Red indicates an upregulation difference, and blue indicates a downregulation difference.

The danazol-induced model group exhibited decreased levels of IL-4 and IL-10 and increased levels of IL-6, IL-17, and TNF-α, across the serum, gonads, and hypothalamus, compared to the control group. In contrast, treatment with both low and high doses of luteolin, as well as triptorelin, led to IL-4 and IL-10 upregulation and IL-6, IL-17, and TNF-α downregulation in all three tissues. These results suggest that danazol-induced CPP is associated with a systemic inflammatory state characterized by suppressed anti-inflammatory and high pro-inflammatory cytokines. Luteolin and triptorelin both reversed this imbalance, with high-dose luteolin demonstrating a stronger anti-inflammatory effect than the low dose, suggesting a clear dose-response relationship (**Figure 8**).

### 3.7 Toll-like receptor signaling pathway modulation by luteolin

**Figure 9:**
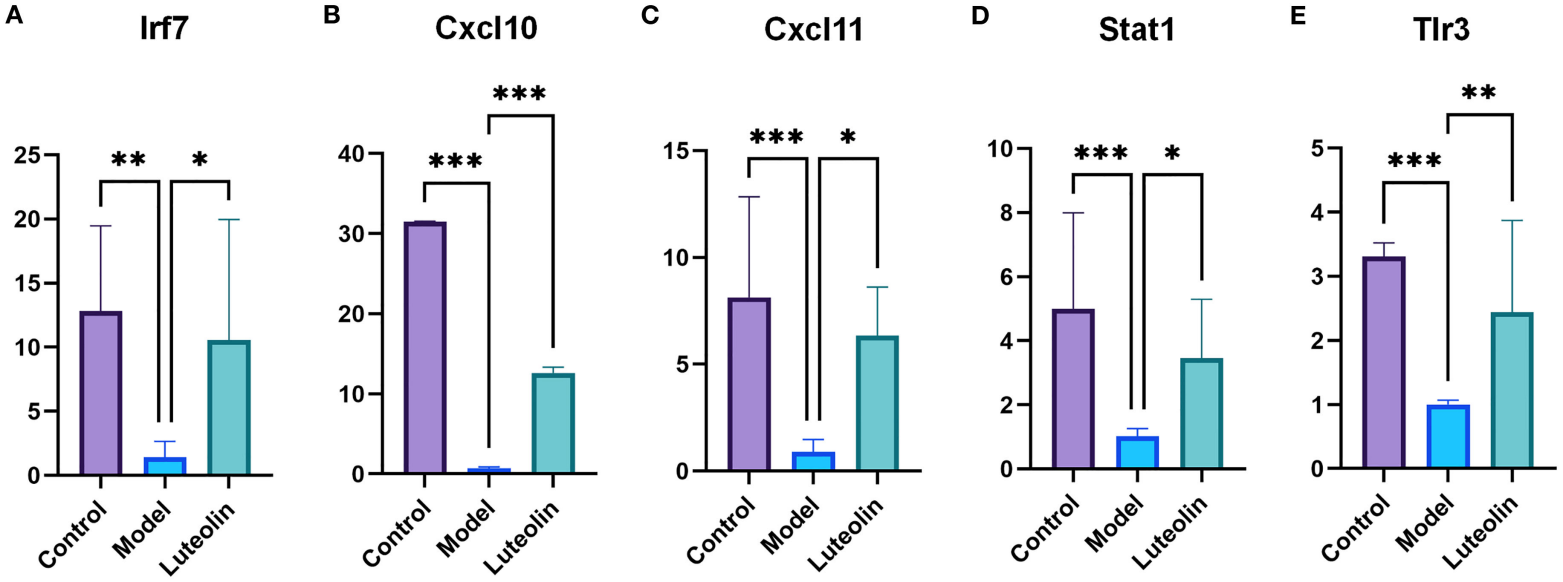
The expression levels of five genes in the hypothalamus of CPP rats were detected using the RT-qPCR method in the control group, model group, and luteolin group. **(A)** RT-qPCR results for Irf7. **(B)** RT-qPCR results for Cxcl10. **(C)** RT-qPCR results for Cxcl11. **(D)** RT-qPCR results for Stat1. **(E)** RT-qPCR results for Tlr3. *P<0.05; **P<0.01; ***P<0.001.

To further validate our findings, we selected several immune-related genes for analysis. RT-qPCR results showed a strong linear correlation with transcriptomic data (**Supplementary Figure 2**), confirming the reliability of the transcriptomic results. The regulatory effects of luteolin and triptorelin on genes associated with the Toll-like receptor signaling pathway were examined by RT-qPCR. Five target genes, Cxcl10, Stat1, Irf7, Cxcl11, and Tlr3, were analyzed. Compared to the control group These genes showed varying degrees of downregulation in the model group. However, treatment with luteolin led to upregulation of all five genes relative to the model group, suggesting that luteolin may restore the expression of key components in this signaling pathway (**Figure 9**).

## 4 Discussion

CPP, marked by the early onset of secondary sexual characteristics, is caused by premature activation of the HPGA and can negatively affect final adult height and psychological well-being (29, 30). TCM presents a promising complementary strategy for CPP management, with potential benefits in modulating endocrine function and enhancing treatment outcomes (18). Both clinical and preclinical studies have demonstrated that TCM, either as monotherapy or in combination with GnRHa, can effectively reduce uterine and ovarian volume, lower serum E2, FSH, and LH levels and delay bone age advancement (12). Network pharmacology analyses have further highlighted key bioactive components in TCM—such as quercetin, β-sitosterol, and luteolin—that target critical signaling pathways including MAPK and PI3K-Akt, providing a mechanistic basis for their therapeutic effects (31). This study explored the therapeutic potential and underlying mechanisms of luteolin, a flavonoid monomer commonly found in TCM formulations for CPP, using a danazol-induced rat model.

The danazol-induced CPP rat model, which mimics accelerated activation of HPGA and early sexual maturation, effectively demonstrated the therapeutic potential of luteolin (19, 31). In this study, triptorelin, a widely used GnRHa was used as the positive control (32, 33). To evaluate luteolin’s efficacy in treating CPP, rats were administered luteolin at 60 and 100 mg/kg doses, which significantly delayed vaginal opening, reduced uterine and ovarian weights and coefficients, and lowered serum E2, LH, and FSH levels. These phenotypic improvements are consistent with effects previously reported for TCM formulations (34, 35). Notably, luteolin exhibited therapeutic efficacy comparable to that of triptorelin across these key parameters. Histological analysis via H&E staining further confirmed that both luteolin and triptorelin suppressed uterine wall thickening and follicular maturation.

To elucidate the underlying mechanisms of luteolin therapeutic effects, both network pharmacology and hypothalamic transcriptomic analyses were conducted. Network pharmacology identified 59 common targets of luteolin associated with CPP, many of which are closely related to inflammatory processes, innate immunity, and adaptive immune responses. RNA sequencing revealed substantial differential gene expression between the luteolin-treated and model groups, as well as between the control and model groups. GO enrichment analysis highlighted significant involvement in biological processes such as inflammatory response, chemokine activity, innate immune response, and antigen processing and presentation. Consistent with these findings, KEGG pathway analysis showed enrichment in pathways related to cytokine–cytokine receptor interaction, chemokine signaling, antigen processing and presentation, and apoptosis. Critically, this integrated multi-omics approach converged on immune dysregulation, strongly implicating the Toll-like receptor signaling pathway as a central mechanism in luteolin’s therapeutic action against CPP. Previous studies have demonstrated a link between PP and aberrant immune function, particularly through the heightened release of pro-inflammatory cytokines—findings that align with our results (36–38). Additionally, various studies have reported on luteolin’s anti-inflammatory and neuroprotective properties, further supporting its potential role in modulating neuroimmune pathways implicated in CPP (39–41).

Experimental validation further supported the immune-inflammatory mechanism of luteolin action. ELISA assays revealed that luteolin significantly decreased the levels of IL-6, IL-17, TNF-α (pro-inflammatory cytokines) and increased the levels of IL-4 and IL-10 (anti-inflammatory cytokines) in the hypothalamus, serum, and ovarian tissues. Importantly, RT-qPCR analysis confirmed the activation of the Toll-like receptor signaling pathway as predicted by transcriptomic data. Luteolin significantly upregulated the expression of Tlr3 in the hypothalamus, which further enhanced the expression of Irf7 and Stat1 transcription factors, ultimately leading to increased levels of effector chemokines Cxcl10 and Cxcl11. Molecular docking results further validated these findings, demonstrating stable binding of luteolin to Cxcl10, Cxcl11, Stat1, Tlr3, and Irf7. Trl3, located within the endosomal compartment of dendritic cells, is essential for immune function and has been linked to multiple pathological conditions, including infections, cancer, autoimmune diseases, and allergies (42). IRF7 plays diverse and multifunctional roles in various biological processes and is linked to inflammation, androgen secretion, and endometrial immune regulation (43). Cxcl10 and Cxcl11 are chemokines implicated not only in immune disorders but also in cancer progression and T cell-mediated immune responses (44). Notably, recent studies have demonstrated that Cxcl10 can disrupt gap junction protein alpha 1 (GJA1) homeostasis between oocytes and granulosa cells, thereby impairing follicular development and ovulation (45). These findings delineate a novel mechanistic cascade wherein luteolin exerts its therapeutic effect against CPP through activation of the Tlr3/Irf7/Stat1/Cxcl10– Cxcl11 axis. Although transcriptomic data suggested possible involvement of other inflammatory pathways, like IL-17 and TNF signaling, the Toll-like receptor pathway emerged as the predominant and experimentally validated mechanism. This discovery is particularly significant in light of emerging evidence linking hypothalamic inflammation to premature activation of the HPGA in CPP pathogenesis (7, 8).

The study has several limitations. Although the danazol-induced rat model is valuable for mimicking aspects of CPP, it may fall short in capturing the complex etiology and heterogeneity of human CPP. Future investigations should employ more targeted *in vitro* and *in vivo* approaches—such as gene knockout/knock-in models and specific pathway modulators to delineate the precise roles of key targets within the Tlr3 signaling axis. Additionally, well-designed clinical trials are essential to assess the safety, efficacy, and long-term effects of luteolin as a therapeutic or adjunctive agent for CPP. Given its natural origin and multifaceted pharmacological properties, luteolin presents a promising candidate for development into nutraceuticals or refined TCM-based interventions. Nevertheless, despite the encouraging findings, substantial further validation is required before luteolin-based therapies for CPP can be considered for clinical application.

## 5 Conclusion

This study presents compelling evidence that luteolin effectively ameliorates CPP in a danazol-induced rat model. Luteolin treatment significantly delayed vaginal opening, reduced uterine and ovarian weights and coefficients, and suppressed key reproductive hormone levels (LH, FSH, and E2) in the serum. Through an integrated approach combining network pharmacology and transcriptomics, followed by targeted experimental validation, we identified the suppression of immune-inflammatory signaling, particularly via the Toll-like receptor pathway, as the primary mechanism underlying luteolin’s therapeutic effects. Mechanistic investigations confirmed that luteolin upregulates hypothalamic Tlr3 expression, thereby activating a downstream cascade involving Irf7 and Stat1, and ultimately enhancing the expression of the effector chemokines Cxcl10 and Cxcl11. In parallel, luteolin increased levels of anti-inflammatory cytokines (IL-4, IL-10) across hypothalamic, serum, and ovarian tissues. This work is the first to delineate a Tlr3-mediated anti-inflammatory mechanism of luteolin in the context of CPP, providing critical insight into its pharmacological actions and strongly supporting its potential as a novel therapeutic candidate for CPP treatment.

## Data Availability

The datasets presented in this study can be found in online repositories. The names of the repository/repositories and accession number(s) can be found in the article/**Supplementary Material**.

## Supplementary material

Supplementary Figure 1 Luteolin 2D Structure. PubChem Compound Summary for CID 5280445, Luteolin. Retrieved August 24, 2025 from https://pubchem.ncbi.nlm.nih.gov/compound/Luteolin.

Supplementary Figure 2 The graph of linear correlation between RT-qPCR results and transcriptomics results. **(A)** is the results of the luteolin group. **(B)** is the results of the model group. R^2^ stands for coefficient of determination.

## References

[1] Bräuner EV Busch AS Eckert-Lind C Koch T Hickey M Juul A. Trends in the incidence of central precocious puberty and normal variant puberty among children in Denmark, 1998 to 2017. JAMA Netw Open. (2020) 3:e2015665. doi: 10.1001/jamanetworkopen.2020.15665, PMID: 33044548 PMC7550972

[2] Nguyen NN Do TD Truong HH Mai AN Chen YC. Difference in precocious puberty between pre-COVID-19 and COVID-19 periods: a meta-analysis. Am J Epidemiol. (2025) 194:1131–9. doi: 10.1093/aje/kwae295, PMID: 39168833

[3] Bradley SH Lawrence N Steele C Mohamed Z. Precocious puberty. Bmj. (2020) 368:l6597. doi: 10.1136/bmj.l6597, PMID: 31932347

[4] Sun Y Liu H Mu C Liu P Hao C Xin Y. Early puberty: a review on its role as a risk factor for metabolic and mental disorders. Front Pediatr. (2024) 12:1326864. doi: 10.3389/fped.2024.1326864, PMID: 39328587 PMC11424421

[5] Lee PA Fuqua JS. Abnormal puberty revisited: A practical approach. Endocrinol Metab Clin North Am. (2024) 53:xi–xii. doi: 10.1016/j.ecl.2024.03.002, PMID: 38677874

[6] Poon K. Behavioral feeding circuit: dietary fat-induced effects of inflammatory mediators in the hypothalamus. Front Endocrinol (Lausanne). (2020) 11:591559. doi: 10.3389/fendo.2020.591559, PMID: 33324346 PMC7726204

[7] Tzounakou AM Stathori G Paltoglou G Valsamakis G Mastorakos G Vlahos NF. Childhood obesity, hypothalamic inflammation, and the onset of puberty: A narrative review. Nutrients 16. (2024) 16:1720. doi: 10.3390/nu16111720, PMID: 38892653 PMC11175006

[8] Valsamakis G Arapaki A Balafoutas D Charmandari E Vlahos NF. Diet-induced hypothalamic inflammation, phoenixin, and subsequent precocious puberty. Nutrients. (2021) 13:3460. doi: 10.3390/nu13103460, PMID: 34684462 PMC8540795

[9] Zevin EL Eugster EA. Central precocious puberty: a review of diagnosis, treatment, and outcomes. Lancet Child Adolesc Health. (2023) 7:886–96. doi: 10.1016/S2352-4642(23)00237-7, PMID: 37973253

[10] Cheuiche AV da Silveira LG de Paula LCP Lucena IRS Silveiro SP. Diagnosis and management of precocious sexual maturation: an updated review. Eur J Pediatr. (2021) 180:3073–87. doi: 10.1007/s00431-021-04022-1, PMID: 33745030

[11] Han XX Zhao FY Gu KR Wang GP Zhang J Tao R. Development of precocious puberty in children: Surmised medicinal plant treatment. BioMed Pharmacother. (2022) 156:113907. doi: 10.1016/j.biopha.2022.113907, PMID: 36411607

[12] Ma Y Sun F Zhang E Li J Yue S Fu Y. Efficacy and mechanism of nourishing yin and purging fire therapy for central precocious puberty based on meta-analysis and network pharmacology. Med (Baltimore). (2023) 102:e36395. doi: 10.1097/MD.0000000000036395, PMID: 38050263 PMC10695624

[13] Zhang J Yang Q Wulu J Zhang Z. Integrated multicomponent analysis based on ultra-high-performance liquid chromatography coupled with quadrupole-Exactive Orbitrap mass spectrometry and network pharmacology to elucidate the effective constituents and potential mechanism of Zhibai Dihuang pill in treating childhood precocious puberty. Rapid Commun Mass Spectrom. (2024) 38:e9831. doi: 10.1002/rcm.9831, PMID: 38837506

[14] Zhang Y Sun N Zhang M Ding Q Wang Q Liang Y. Effects of fuyou formula on gnRH secretion and related gene expression in treating precocious puberty. Front Pharmacol. (2022) 13:852550. doi: 10.3389/fphar.2022.852550, PMID: 35359850 PMC8962374

[15] Wan C Liang Q Ma Y Wang Y Sun L Lai J. Luteolin: a natural product with multiple mechanisms for atherosclerosis. Front Pharmacol. (2025) 16:1503832. doi: 10.3389/fphar.2025.1503832, PMID: 40213687 PMC11983452

[16] Guo C Sun N Hu K Bai G Zhang M Wang Q. Integrated pharmacological analysis on the mechanism of fuyou formula in treating precocious puberty. Front Pharmacol. (2021) 12:649732. doi: 10.3389/fphar.2021.649732, PMID: 34025416 PMC8138182

[17] Zhu M Sun Y Su Y Guan W Wang Y Han J. Luteolin: A promising multifunctional natural flavonoid for human diseases. Phytother Res. (2024) 38:3417–43. doi: 10.1002/ptr.8217, PMID: 38666435

[18] Nguyen NN Lin CY Tsai WL Huang HY Chen CM Tung YT. Natural sweetener glycyrrhizin protects against precocious puberty by modulating the gut microbiome. Life Sci. (2024) 350:122789. doi: 10.1016/j.lfs.2024.122789, PMID: 38848942

[19] Sun Y Perry GN Yu J Chen B Tian Z. Effect of nourishing “Yin”-removing “Fire” Chinese herbal mixture on hypothalamic kisspeptin expression in female precocious rats. J Ethnopharmacol. (2010) 127:274–9. doi: 10.1016/j.jep.2009.11.009, PMID: 19931369

[20] Tan J Zhang J Yang W Li J Zang Y Yang S. Integrated transcriptomics and network pharmacology to reveal the mechanism of Physochlainae Radix in the treatment of asthma. Phytomedicine. (2025) 139:156470. doi: 10.1016/j.phymed.2025.156470, PMID: 39947003

[21] Zhao C Bai X Ding Y Wen A Fu Q. Combining systems pharmacology, metabolomics, and transcriptomics to reveal the mechanism of Salvia miltiorrhiza-Cortex moutan herb pair for the treatment of ischemic stroke. Front Pharmacol. (2024) 15:1431692. doi: 10.3389/fphar.2024.1431692, PMID: 39314757 PMC11417465

[22] Zhou F Mao J Jin Z Zhu L Li X. Multi-omic analysis of precocious puberty girls: pathway changes and metabolite validation. Front Endocrinol (Lausanne). (2024) 15:1285666. doi: 10.3389/fendo.2024.1285666, PMID: 38487340 PMC10937432

[23] Morris GM Huey R Olson AJ. Using AutoDock for ligand-receptor docking. Curr Protoc Bioinf Chapter. (2008) 8:14s24. doi: 10.1002/0471250953.bi0814s24, PMID: 19085980

[24] Wang Y Bryant SH Cheng T Wang J Gindulyte A Shoemaker BA. PubChem bioAssay: 2017 update. Nucleic Acids Res. (2017) 45:D955–d963. doi: 10.1093/nar/gkw1118, PMID: 27899599 PMC5210581

[25] Young MD Wakefield MJ Smyth GK Oshlack A. Gene ontology analysis for RNA-seq: accounting for selection bias. Genome Biol. (2010) 11:R14. doi: 10.1186/gb-2010-11-2-r14, PMID: 20132535 PMC2872874

[26] Kanehisa M Araki M Goto S Hattori M Hirakawa M Itoh M. KEGG for linking genomes to life and the environment. Nucleic Acids Res. (2008) 36:D480–4. doi: 10.1093/nar/gkm882, PMID: 18077471 PMC2238879

[27] Trapnell C Williams BA Pertea G Mortazavi A Kwan G van Baren MJ. Transcript assembly and quantification by RNA-Seq reveals unannotated transcripts and isoform switching during cell differentiation. Nat Biotechnol. (2010) 28:511–5. doi: 10.1038/nbt.1621, PMID: 20436464 PMC3146043

[28] Harshitha R Arunraj DR Real-time quantitative PCR. A tool for absolute and relative quantification. Biochem Mol Biol Educ. (2021) 49:800–12. doi: 10.1002/bmb.21552, PMID: 34132460

[29] Kim SJ Kim JH Hong YH Chung IH Lee EB Kang E. 2022 Clinical practice guidelines for central precocious puberty of Korean children and adolescents. Ann Pediatr Endocrinol Metab. (2023) 28:168–77. doi: 10.6065/apem.2346168.084, PMID: 37798893 PMC10556443

[30] Micangeli G Paparella R Tarani F Menghi M Ferraguti G Carlomagno F. Clinical management and therapy of precocious puberty in the sapienza university pediatrics hospital of rome, Italy. Children (Basel). (2023) 10:1672. doi: 10.3390/children10101672, PMID: 37892335 PMC10604951

[31] Chen X Zheng M Fei X Ma X. Analysis of the efficacy of Dabuyin pill combined with gonadotropin-releasing hormone analogue in the treatment of central precocious puberty girls based on network pharmacology. Transl Pediatr. (2023) 12:364–74. doi: 10.21037/tp-23-111, PMID: 37035395 PMC10080485

[32] Bertelloni S Mucaria C Baroncelli GI Peroni D. Triptorelin depot for the treatment of children 2 years and older with central precocious puberty. Expert Rev Clin Pharmacol. (2018) 11:659–67. doi: 10.1080/17512433.2018.1494569, PMID: 29957076

[33] Valenzise M Nasso C Scarfone A Rottura M Cafarella G Pallio G. Leuprolide and triptorelin treatment in children with idiopathic central precocious puberty: an efficacy/tolerability comparison study. Front Pediatr. (2023) 11:1170025. doi: 10.3389/fped.2023.1170025, PMID: 37266535 PMC10229807

[34] Bai GL Hu KL Huan Y Wang X Lei L Zhang M. The traditional chinese medicine fuyou formula alleviates precocious puberty by inhibiting GPR54/gnRH in the hypothalamus. Front Pharmacol. (2020) 11:596525. doi: 10.3389/fphar.2020.596525, PMID: 33551803 PMC7859969

[35] Zhou L Ren Y Li D Zhou W Li C Wang Q. Timosaponin AIII attenuates precocious puberty in mice through downregulating the hypothalamic-pituitary-gonadal axis. Acta Biochim Pol. (2023) 70:183–90. doi: 10.18388/abp.2020_6450, PMID: 36928746

[36] Delany FM Byrne ML Whittle S Simmons JG Olsson C Mundy LK. Depression, immune function, and early adrenarche in children. Psychoneuroendocrinology. (2016) 63:228–34. doi: 10.1016/j.psyneuen.2015.10.003, PMID: 26492635

[37] Shokri B Heidarianpour A Shokri E. Effect of exercise and detraining on signs of puberty and selected inflammatory markers in girls with precocious puberty. Med Sci Sports Exerc. (2023) 55:1133–42. doi: 10.1249/MSS.0000000000003138, PMID: 36790953

[38] Wang T Liu W Wang C Ma X Akhtar MF Li Y. MRKNs: gene, functions, and role in disease and infection. Front Oncol. (2022) 12:862206. doi: 10.3389/fonc.2022.862206, PMID: 35463379 PMC9024132

[39] Aziz N Kim MY Cho JY. Anti-inflammatory effects of luteolin: A review of *in vitro*, *in vivo*, and in silico studies. J Ethnopharmacol. (2018) 225:342–58. doi: 10.1016/j.jep.2018.05.019, PMID: 29801717

[40] Gendrisch F Esser PR Schempp CM Wölfle U. Luteolin as a modulator of skin aging and inflammation. Biofactors. (2021) 47:170–80. doi: 10.1002/biof.1699, PMID: 33368702

[41] Nabavi SF Braidy N Gortzi O Sobarzo-Sanchez E Daglia M Skalicka-Woźniak K. Luteolin as an anti-inflammatory and neuroprotective agent: A brief review. Brain Res Bull. (2015) 119:1–11. doi: 10.1016/j.brainresbull.2015.09.002, PMID: 26361743

[42] Hsieh ML Nishizaki D Adashek JJ Kato S Kurzrock R. Toll-like receptor 3: a double-edged sword. biomark Res. (2025) 13:32. doi: 10.1186/s40364-025-00739-5, PMID: 39988665 PMC11849352

[43] Hu M Zhang Y Li X Cui P Sferruzzi-Perri AN Brännström M. TLR4-associated IRF-7 and NFκB signaling act as a molecular link between androgen and metformin activities and cytokine synthesis in the PCOS endometrium. J Clin Endocrinol Metab. (2021) 106:1022–40. doi: 10.1210/clinem/dgaa951, PMID: 33382900

[44] Li X Lu M Yuan M Ye J Zhang W Xu L. CXCL10-armed oncolytic adenovirus promotes tumor-infiltrating T-cell chemotaxis to enhance anti-PD-1 therapy. Oncoimmunology. (2022) 11:2118210. doi: 10.1080/2162402X.2022.2118210, PMID: 36092638 PMC9450898

[45] Zhao M Liao B Yun C Qi X Pang Y. Liraglutide improves follicle development in polycystic ovary syndrome by inhibiting CXCL10 secretion. Reprod Biol Endocrinol. (2024) 22:98. doi: 10.1186/s12958-024-01269-9, PMID: 39107809 PMC11302332

