# Supplementary material for: Therapeutic potential of luteolin in central precocious puberty: insights from a danazol-induced rat model
Source: Front Endocrinol (Lausanne). 2025 Sep 12;16:1666932. doi: 10.3389/fendo.2025.1666932 (PMC12463909; doi:10.3389/fendo.2025.1666932)
Supplement: Supplementary Figure 1 — Luteolin 2D Structure. PubChem Compound Summary for CID 5280445, Luteolin. Retrieved August 24, 2025 from https://pubchem.ncbi.nlm.nih.gov/compound/Luteolin. [file DataSheet1.docx]

Supplementary Material

# Supplementary Data

The transcriptome data has been uploaded to the NCBI public database, with the accession number: PRJNA1286934. Other source data and original images files are uploaded to Jianguoyun (https://www.jianguoyun.com/), and the folder is named "Raw Data". The specific sharing link is as follows (https://www.jianguoyun.com/p/DSen0yYQ3K_JDRii3IoGIAA).

# Supplementary Figure 1


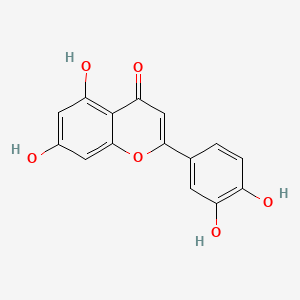


**Supplementary Figure 1.** Luteolin 2D Structure. PubChem Compound Summary for CID 5280445, Luteolin. Retrieved August 24, 2025 from https://pubchem.ncbi.nlm.nih.gov/compound/Luteolin.

# Supplementary Figure 2


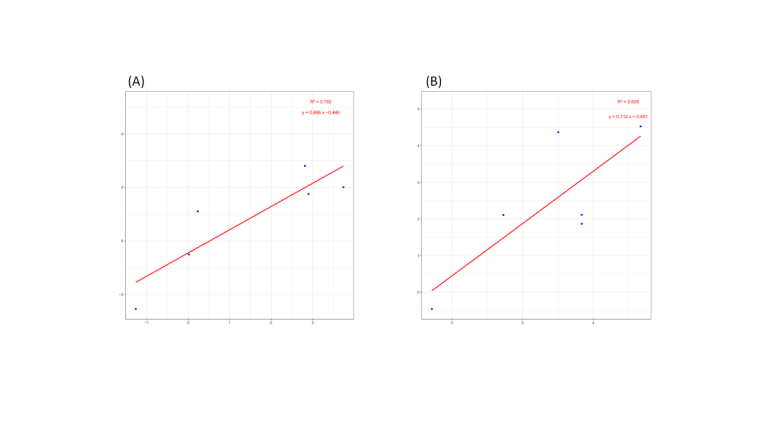


**Supplementary Figure 2.** The graph of linear correlation between RT-qPCR resulyyyyts and transcriptomics results. (A) is the results of the luteolin group. (B) is the results of the model group. R^2^ stands for coefficient of determination.
