# Supplementary figures and images for: Therapeutic potential of luteolin in central precocious puberty: insights from a danazol-induced rat model
Source: Front Endocrinol (Lausanne). 2025 Sep 12;16:1666932. doi: 10.3389/fendo.2025.1666932 (PMC12463909; doi:10.3389/fendo.2025.1666932)

| 1O80 | CXCL10 | 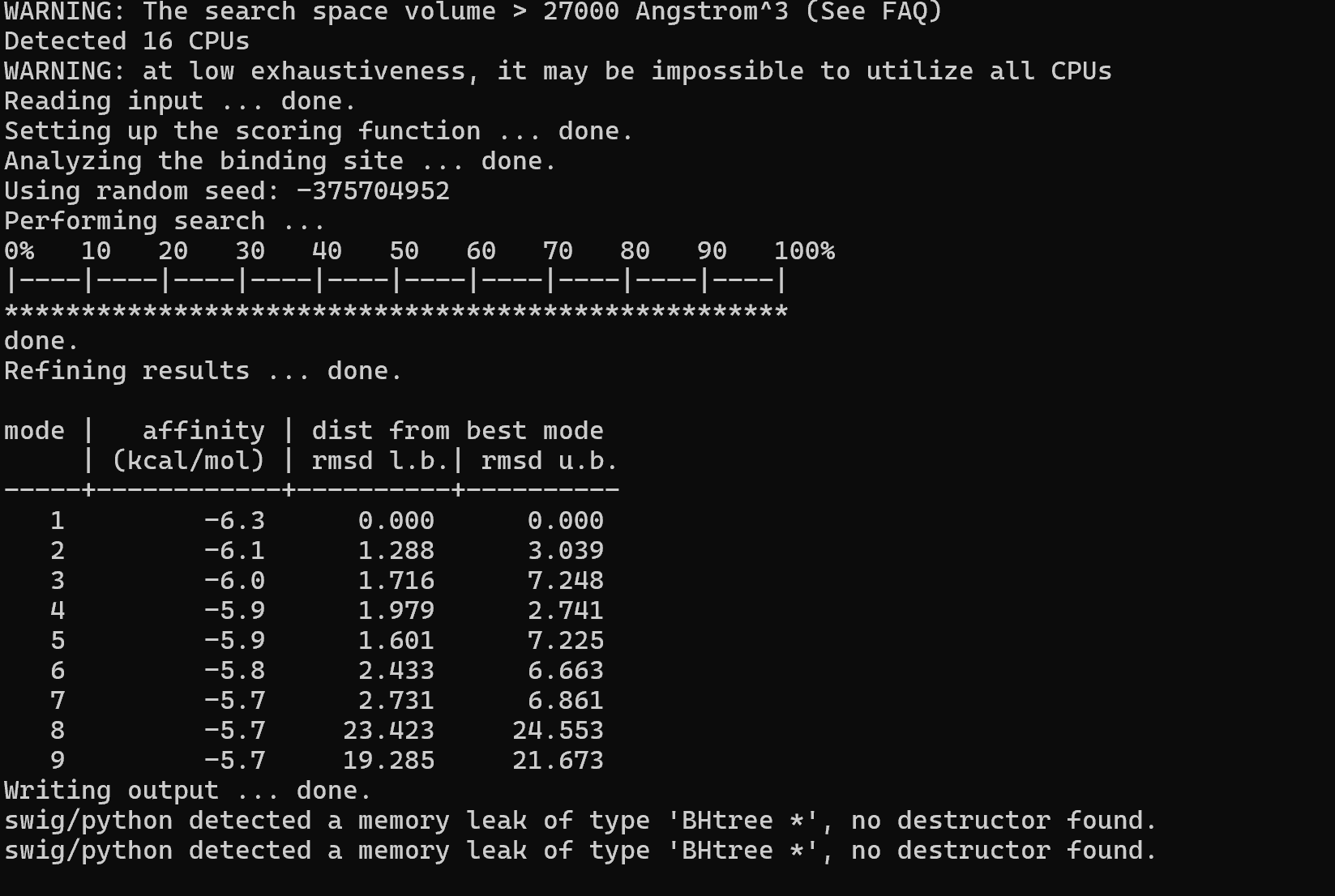 |
| --- | --- | --- |
| 1RJT | CXCL11 | 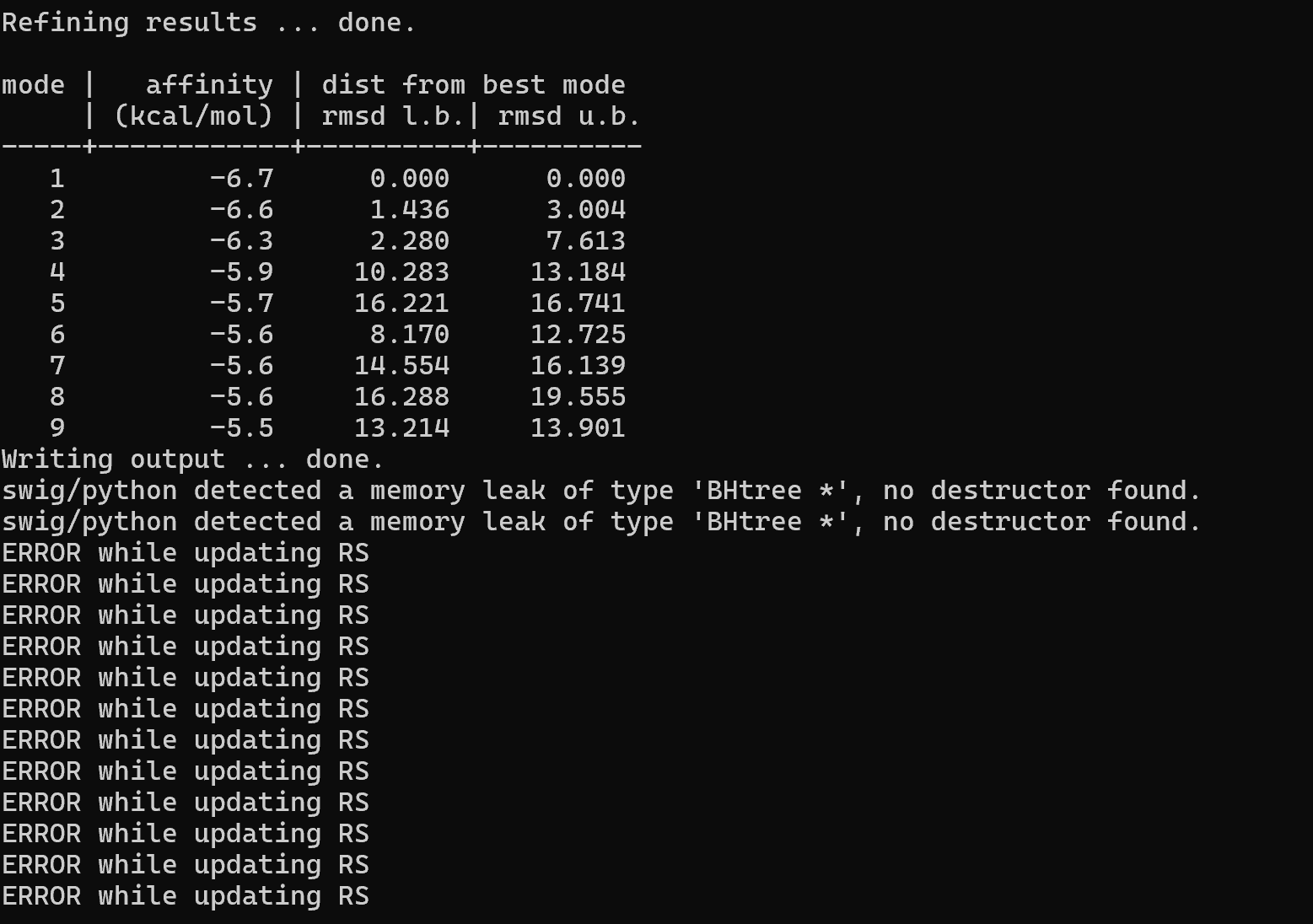 |
| 1YVL | STAT1 | 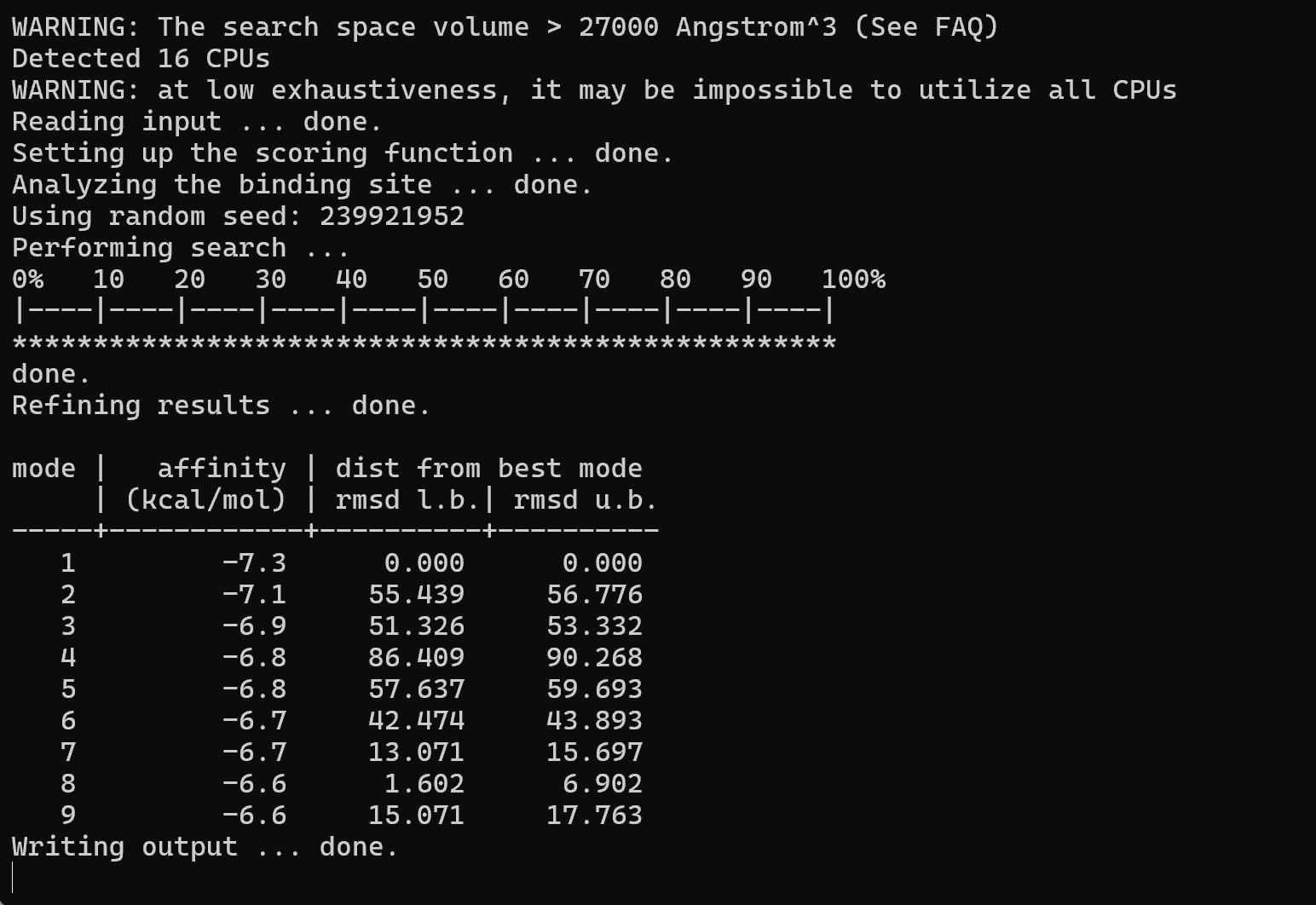 |
| 1ZIW | TLR3 | 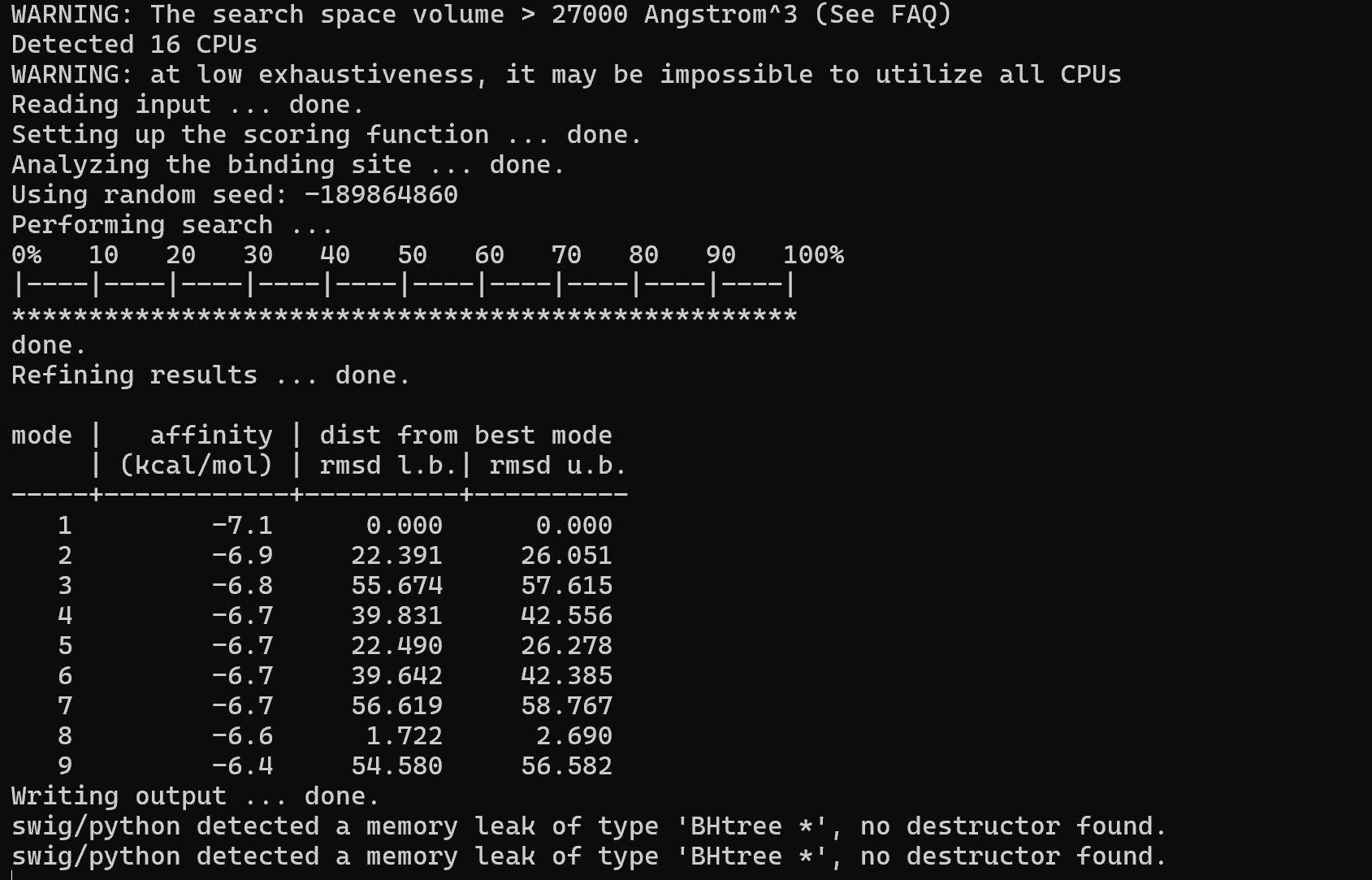 |
| 2O61 | IRF7 | 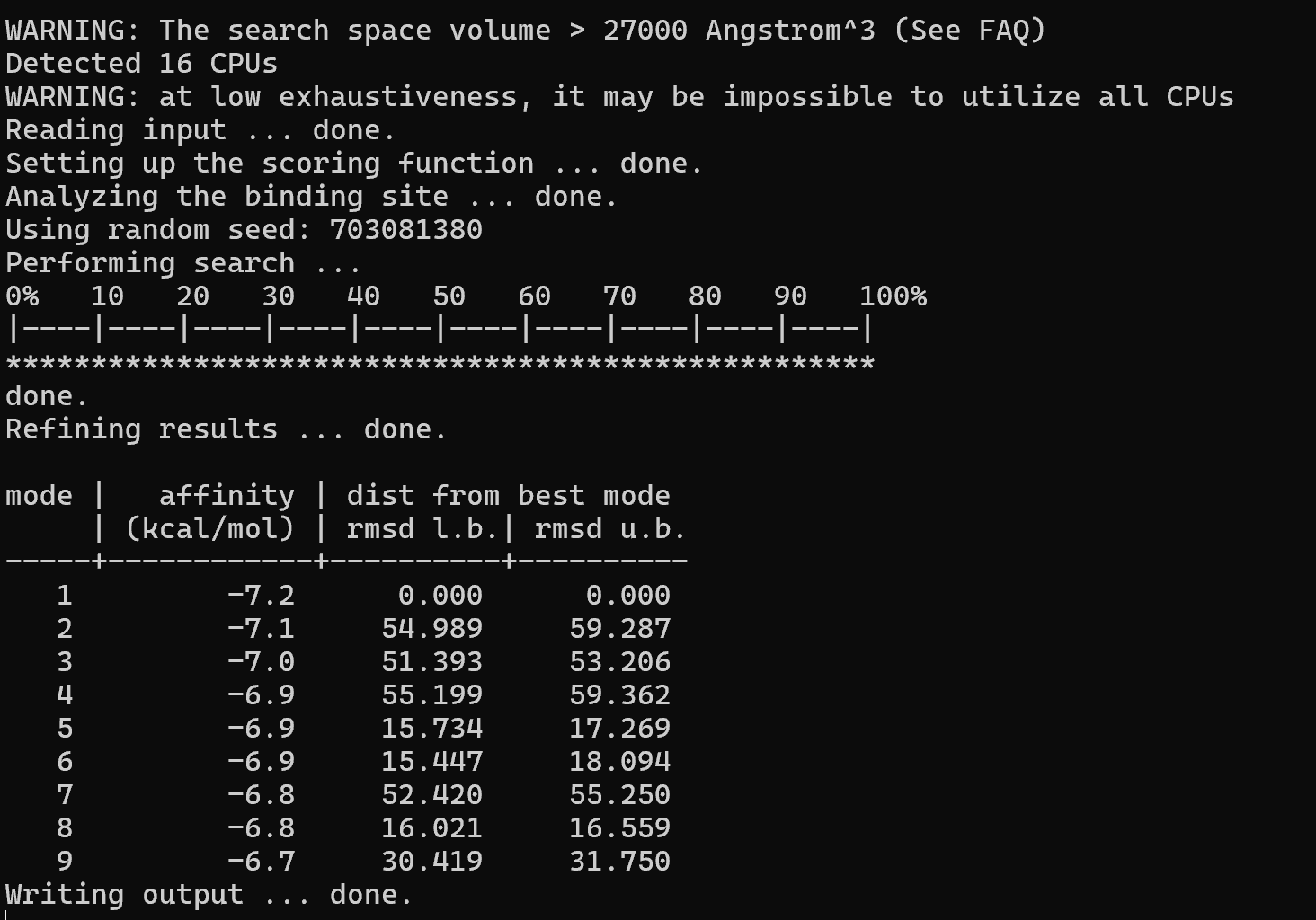 |

Supplement: Supplementary file 6 [file Table5.docx]
